# Supplementary material for: The Role of Gratitude in a Positive Psychology Group Intervention Program Implemented for Undergraduate Engineering Students
Source: Behav Sci (Basel). 2023 Jun 2;13(6):460. doi: 10.3390/bs13060460 (PMC10295172; doi:10.3390/bs13060460)
Supplement: Supplementary file 1 [file behavsci-13-00460-s001.zip › behavsci-2360702-supplementary.pdf]

Supplementary Material

Supplementary Data

Table S1. Measures administration.

**Administration**	Pre-test 1st week	3rd & 4th session	Post-test 5th week		Pre-test 1st week	3rd & 4th week	Post-test 5th week
**Experimental group**	mDES	GQ-6	mDES	**Control group**	mDES	GQ-6	mDES
	CD-RISC		CD-RISC		CD-RISC		CD-RISC
	SHS		SHS		SHS		SHS
	LOT-R		LOT-R		LOT-R		LOT-R

Note: Pre-test: Pre-intervention, Post-test: Post-intervention, mDES: Differential Emotions Scale-modified, CD-RISC: Connor-Davidson Resilience Scale, SHS: Subjective Happiness Scale, LOT-R: Revised Life Orientation Test, GQ-6: Gratitude Questionnaire-six item form.

Table S2. Means, standard deviations, and pre and post-intervention between-groups and within-groups differences in gratitude.

**Variable**	**Experimental group**				**Control group**				**Independent t-test**	
	M	*SD*			*M*	*SD*			*t*	*p*
Pre-test gratitude	5.09	.88			4.98	.78			.54	.59
	**Experimental group**				**Control group**					
			**Paired t-test**				**Paired t-test**		**Independent t-test**	
	M	*SD*	*t*	*p*	*M*	*SD*	*t*	*p*	*t*	*p*
Post-test gratitude	5.53	.98	-3.39	.002	4.88	.85	1.14	.26	2.98	.004

Note: ns: non-significant, Pre-test: Pre-intervention, Post-test: Post-intervention, GQ-6: Gratitude Questionnaire-six item form.

Table S3. Correlations between gratitude and the other psychological variables and multivariate regression analysis.

**Predictive variable**	**Post-test outcome variables**	***r***	***F*(1, 18)**	η_p^2	***p***
Post-test gratitude	Positive emotions	.60***	1.46	.64	.230
	Negative emotions	-.28ns	1.16	.58	.390

	Psychological resilience	.63***	2.16	.72	.070
	Subjective happiness	.69***	3.45	.81	.009
	Optimism	.51**	2.47	.75	.042

Note: ** *p* < .01; *** *p* < .001; ns: non-significant; Post-test: Post-intervention.
